# The Sound of Dinner

**DOI:** 10.1371/journal.pbio.0040107

**Published:** 2006-03-07

**Authors:** Françoise Chanut

Bats are not blind, but many rely primarily on sounds to navigate and forage. For the big brown bat, a common denizen of treetops and old house attics, dinner starts at dusk and consists of flying insects—mostly beetles—caught on the wing. As they soar from their caches, bats probe the night with piercing ultrasonic cries, listening for guidance cues in the echoes returned by reflective surfaces. In a wide-open space, the cries dissipate before they bump into the surroundings, and the only perceptible echoes come from objects—potentially winged insects—that pass within a few yards of the bat's large ears. But in a more complex environment, such as a wooded field, a bat's challenge is to hear the faint echo of a tasty morsel over the noise bouncing off trunks, leaves, and every blade of grass.

In a recent study, Cynthia Moss and her colleagues detected rhythm changes in the cries of bats hunting in cluttered versus open environments. The researchers' observations show that bats have an uncanny ability to adjust their sensory apparatus—in this case, their vocal performance—to their perception of the environment, a phenomenon known as active listening.

Big brown bats can emit cries of variable pitch, length, and repetition rate, which generate a wealth of details for their finely tuned ears to ponder in the returning sounds: an echo with a sliding pitch suggests a moving object, while intensity flickers signal a fluttering wing. Subtle discrepancies in pitch spectrum reveal an object's size and texture, while time delays between cry and echo translate into distance. Having thus identified an appetizing prey, the bat zeroes in on it with cries of increasing frequency and decreasing duration. Its final pounce is guided by rapid spitfire known as the final buzz.

Moss and her colleagues focused on the last few seconds before the final buzz, a time when bats emit clusters of two or more short pulses separated by silent intervals ten to 25 milliseconds long. The researchers hypothesized that these quick, repetitive pulses, known as “sonar strobe groups,” might be particularly well suited to resolving a small object from a complex background. They filmed and recorded individual big brown bats launching on a tethered mealworm inside a dark, bedroom-sized chamber. A synthetic plant hanging at various distances from the worm created clutter. Simultaneous sound and high-speed video recordings of the attack allowed the researchers to match cries and echoes precisely with the stages of the hunt and the bats' wing-beat pattern.

In an empty chamber, bats attempted capture and succeeded every time, after a mere two-second hunt. But clutter muddied their hearing. When the worm hung closest to the plant (ten centimeters), the bats made for the prey only half of the time, and overwhelmingly failed, presumably because they couldn't distinguish worm from foliage. As the distance between prey and clutter increased to 40 centimeters, attempts and success rates increased while hunting times decreased from one minute back to a few seconds.

Clutter also altered the cries' sequence, most dramatically within the last second of the hunt. Bats curtailed their final buzz 3-fold and spent more time strobing in the presence of clutter, an indication that strobing is indeed used to increase resolution power. In addition, they did not quicken the pace of their strobe groups as fast: the silent intervals between pulses progressively shortened in both situations, but slowly and only to a third of their original length in clutter, whereas they quickly dropped by half in an open-room hunt. Interestingly, bats emitted strobe groups at any stage of flight, contrary to former theories that linked sound production to wing beat.

The slower rhythm change of the sonar strobe groups in clutter is a further hint that the bats use these sound clusters to improve their perception of complex echoes. Still, the fact that there is useful information in a signal does not mean that the information is used. Ultimately, experiments that disrupt the strobe groups will be needed to prove their utility.

## 

**Figure:**
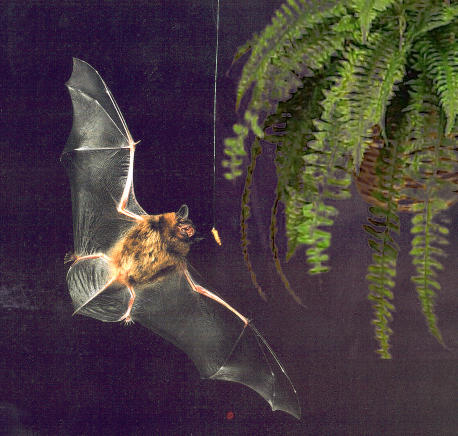
Echolocating bats adjust their vocalizations to catch insects against a changing environmental background.

